# Mechanisms and Optimization Strategies of Paracrine Exosomes from Mesenchymal Stem Cells in Ischemic Heart Disease

**DOI:** 10.1155/2023/6500831

**Published:** 2023-11-22

**Authors:** Xiaorong Yin, Lizhi Lin, Fang Fang, Bin Zhang, Cheng Shen

**Affiliations:** ^1^Department of Clinical Medicine, Affiliated Hospital of Jining Medical University, Jining Medical University, Jining, Shandong, China; ^2^Department of Cardiology, Jining Key Laboratory for Diagnosis and Treatment of Cardiovascular Diseases, Affiliated Hospital of Jining Medical University, Jining, Shandong, China; ^3^Department of Laboratory Medicine, Affiliated Hospital of Jining Medical University, Jining Medical University, Jining, Shandong, China; ^4^Shandong University of Traditional Chinese Medicine, Jinan, Shandong, China

## Abstract

The morbidity and mortality of myocardial infarction (MI) are increasing worldwide. Mesenchymal stem cells (MSCs) are multipotent stem cells with self-renewal and differentiation capabilities that are essential in tissue healing and regenerative medicine. However, the low implantation and survival rates of transplanted cells hinder the widespread clinical use of stem cells. Exosomes are naturally occurring nanovesicles that are secreted by cells and promote the repair of cardiac function by transporting noncoding RNA and protein. In recent years, MSC-derived exosomes have been promising cell-free treatment tools for improving cardiac function and reversing cardiac remodeling. This review describes the biological properties and therapeutic potential of exosomes and summarizes some engineering approaches for exosomes optimization to enhance the targeting and therapeutic efficacy of exosomes in MI.

## 1. Introduction

Myocardial infarction (MI) involves the irreversible death of cardiomyocytes due to prolonged oxygen deprivation by an obstructed blood supply (ischemia), which results in contractile dysfunction and cardiac remodeling. Although significant progress has been made in medical treatments, such as thrombolytic therapy, percutaneous coronary intervention, and coronary artery bypass grafting surgery, MI is still the leading cause of cardiovascular disease (CVD)-associated death [1]. The high prevalence and mortality of MI suggest that it is essential to continue the search for suitable treatments. Stem cells have powerful regenerative potential and are expected to be some of the best candidates for MI treatment [2, 3]. Mesenchymal stem cells (MSCs) are multipotent stem cells that can self-renew and differentiate. They have received much attention due to their strong proliferative capacity, multidirectional differentiation potential, immunomodulatory properties, low immunogenicity, and ease of isolation and expansion. MSCs may protect the myocardium by reducing inflammation, promoting angiogenesis around infarcted areas, increasing resistance to apoptosis, and inhibiting fibrosis [4]. In recent years, some pretreatment methods, such as hypoxia pretreatment, drug pretreatment, cytokine pretreatment, and gene modification, have been used to enhance the functional benefits of transplanted stem cells to promote cardiac regeneration and angiogenesis and inhibit fibrosis progression. However, poor engraftment and survival rates of MSCs in the myocardium inhibit their therapeutic efficacy. Exosomes, which are products of MSCs, have been prioritized in acellular therapy for cardiac rehabilitation after MI and other infections, and they may yield significant economic and social benefits [5]. Numerous studies have indicated that MSC-derived exosomes (MSC-Exos) can promote cardiomyocyte survival and angiogenesis, inhibit fibrosis, and regulate the cardiac microenvironment, which are indispensable therapeutic strategies for alleviating ischemic heart disease [6–8]. Exosome targeting has also been used to enhance the capability of exosomes to target homologous receptors in specific tissues and organs [7]. MSC-Exos are attractive alternatives for acellular therapy after MI [9]. This review aims to discuss the biological roles and mechanisms of exosomes, as well as the best strategies to improve the efficacy of exosome-based treatment, and reveal the remarkable potential of exosomes in the treatment of MI.

## 2. Biological Properties of MSCs

Currently, the stem cells used for MI therapy include MSCs, embryonic stem cells (ESCs), placental-derived stem cells, and cord blood-derived stem cells. MSCs are multipotent and nonhematopoietic adult stem cells with self-renewal and differentiation abilities [10, 11]. MSCs can be isolated from a very diverse range of tissues or organs, including adipose tissue, bone marrow, the placenta, the umbilical cord blood and umbilical cord, skin, skeletal muscle, tendons, synovial membranes, the endometrium, amniotic fluid, the amniotic membrane, peripheral blood, menstrual blood, salivary glands, pulp, and periodontal ligaments [12–15]. Clinically, MSCs are derived mainly from bone marrow, umbilical cord blood, and adipose tissue [3, 16, 17]. In 2006, the International Society for Cell Therapy proposed the following minimal criteria for defining MSCs: adherent growth in stable culture; CD105, CD73, and CD90 expression but no expression of CD45, CD34, CD14 or CD11b, CD79*α* or CD19, and HLA-DR; and an in vitro ability to differentiate into osteoblasts, adipocytes, and chondrocytes [18]. MSCs are capable of secreting a range of cytokines, such as vascular endothelial growth factor (VEGF) and pigment epithelial-derived factor [19, 20]. Preclinical findings in rat and porcine MI models have indicated that MSCs can significantly reduce MI size, restore myocardial contractility, and improve the structure and function of infarcted hearts through the synergistic effects of myogenesis and angiogenesis [21, 22]. Hare et al. [23] conducted the first clinical trial of MSCs for MI in 2005 and demonstrated the safety of allogeneic human bone marrow-derived MSCs (hBMMSCs) for treating MI. In addition, studies have shown that intravenous, intracoronary, or myocardial injection of autologous or allogeneic MSCs is safe, and beneficial effects have been observed over 12 months of long-term follow-up [24, 25]. While some clinical trials have demonstrated the effectiveness of MSC treatment, some studies have not observed a benefit from MSC treatment [26, 27]. The differential therapeutic effects of MSCs may be related to the method of cell acquisition, the transplantation step, the survival of cells after transplantation, poor homing, and the high dose needed to maintain the therapeutic effect [28–31]. Moreover, numerous studies in recent years have demonstrated that MSCs help restore cardiac function mainly through their paracrine effects, especially those involving exosomes [32, 33]. Exosomes derived from MSCs (MSC-Exos) have the potential to promote cardiomyocyte survival, proliferation, and angiogenesis and limit the inflammatory response, which can be used as a practicable strategy for cell-free heart repair [34, 35]. These biological activities of MSCs provide new ideas for treating CVDs.

## 3. Biological Properties of Paracrine Exosomes from MSCs

Extracellular vesicles (EVs) mediate the communication of cell-to-cell, which are membrane vesicles (MVs) released by cells [36]. EVs contain a range of bioactive substances, including DNA, RNA, lipids, metabolites, and cytosolic and cell-surface proteins [37, 38]. The International Extracellular Vesicle Society classifies EVs into the following categories: exosomes, apoptotic bodies, and MVs [36, 39, 40]. Exosomes are extracellular membranous nanovesicles with sizes ranging from 30 to 150 nm [29, 41]. Exosomes are formed by inward germination of the multivesicular body membrane and subsequent fusion with the plasma membrane to release intraluminal vesicles into the extracellular space [38, 40, 42]. In addition, there is evidence that exosomes germinate directly from plasma membranes [43]. To date, we have found that ultracentrifugation, precipitation, density gradient centrifugation, immune affinity capture, microfluidic technologies, and size-exclusion chromatography can all be used to isolate exosomes [44–47]. The biomarkers of exosomes are heat shock proteins, ALG-2-interacting protein X (Alix), and integral membrane tetraspanin proteins (CD81, CD63, and CD9), which are widespread in all exosomes [48–51]. In addition, the cargo of exosomes includes many biologically active substances, such as proteins, lipids, and genetic material (mRNAs, miRNAs, long noncoding RNAs, and DNA) [52]. The Exo-Carta exosome database (http://www.exocarta.org) is a collection of 9,769 proteins, 3,408 mRNAs, 2,838 miRNAs, and 1,116 lipids identified in exosomes from diverse cell types and different species. Exosomes play a critical role in cell-to-cell communication by delivering biologically active molecules [41, 53–56]. Recently, exosomes have been shown to participate in many processes, including cell survival, angiogenesis, and immune reactions, by altering the communication among cells/organs [55]. Studies have shown that MSC-Exos have significant advantages over their parental cells [57], such as long-term stability, more accessible storage, lower immune rejection, less tumorigenicity, and lower production costs. Moreover, the smaller size of exosomes relative to their parental cells enables them to pass through capillaries without clogging them [29, 58]. Lai et al. [59] confirmed the role of human ESC-derived MSC-Exos in decreasing myocardial fibrosis and enhancing cardiac function for the first time in 2010. Since then, research on MSC-Exos has reached a new zenith. The role of MSC-Exos in cardioprotection has been heavily investigated, laying the groundwork for future treatments of ischemic heart disease.

## 4. The Therapeutic Potential of MSC-Exos in MI

**Figure 1:**
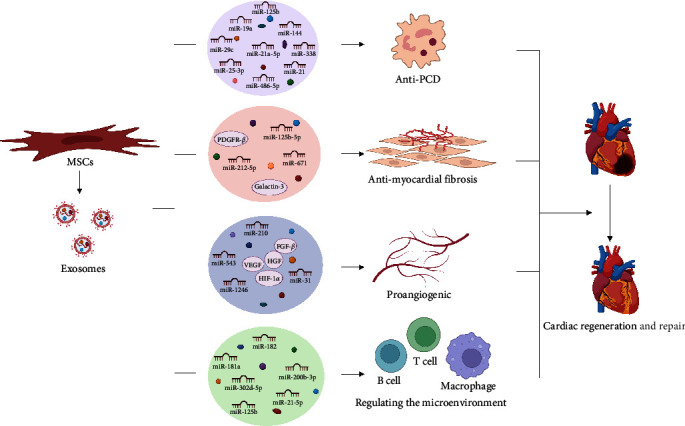
Reparative effect of MSC-Exos on ischemic hearts after MI. After myocardial infarction (MI), the intramuscular injection of exosomes derived from mesenchymal stem cells (MSC-Exos) had an anti-programmed cell death (PCD) effect by releasing miR-19a, miR-144, miR-486-5p, miR-21, miR-21a-5p, miR-25-3p, and other miRNAs. Galectin-3, miR-212-5p, miR-125b-5p, PDGFR-*β*, and miR-671 in MSC-Exos promote ischemic myocardial repair by antimyocardial fibrosis. The hepatocyte growth factor (HGF), angiogenic fibroblast growth factor-*β* (FGF-*β*), vascular endothelial growth factor (VEGF), miR-31, miR-543, and miR-1246 in MSC-Exos can promote the generation of myocardial vascular endothelial cells and maintain myocardial blood flow. Substances such as miR-181a, miR-125b, miR-182, miR-21-5p, miR-302d-5p, and miR-200b-3p released by MSC-Exos can regulate the cardiac microenvironment after myocardial infarction, reduce the inflammatory response and promote myocardial repair.

After MI, the ventricle exhibits a range of responses, such as cardiac hypertrophy, interstitial fibrosis, and inflammation (Figure 1). Programmed cell death (PCD) pathways are activated after ischemia or hypoxia, and they are the major causes of heart failure [45]. Many studies have shown that MSC-Exos can inhibit PCD and fibrosis, promote angiogenesis, and improve the microenvironment of the ischemic myocardium [60, 61]. As some of the most common components of exosomes, microRNAs (miRNAs) play crucial roles in the physiology of the cardiovascular system and CVD progression [62]. Next, we will elaborate on the role of exosomes in MI from several perspectives.

### 4.1. Inhibiting PCD

**Figure 2:**
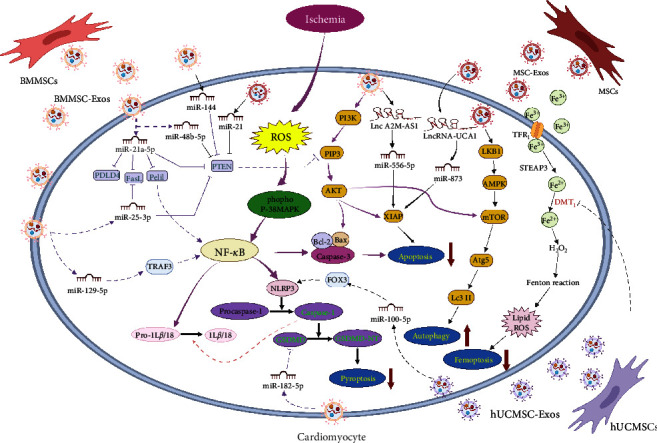
Exosomes from different MSCs mediate PCD in myocardial cells after myocardial infarction. MiR-144 and miR-486-5p in exosomes derived from bone marrow mesenchymal stem cells (BMMSC-Exos) and miR-21 in endometrial exosomes derived from mesenchymal stem cells (MSC-Exos) can inhibit apoptosis of hypoxic cardiomyocytes by targeting the PTEN/PI3K/AKT signaling pathway. MiR-21a-5p and miR-25-3p in BMMSC-Exos can reduce the apoptosis of hypoxic cardiomyocytes by downregulating the expression of proapoptotic genes such as FasL, PDPD4, Peli1, and PTEN. MiR-129-5p in BMMSC-Exos inhibits cardiomyocyte apoptosis by regulating the TRAF3/NF-*κ*B signaling pathway. BMMSC-Exos induce autophagy in cardiomyocytes by activating the AMPK/mTOR and AKT/mTOR signaling pathways. Human BMMSC-Exos inhibit pyroptosis by inhibiting the expression of Caspase-1 and NLRP3. Moreover, miR-182-5p in BMMSC-Exos inhibit pyroptosis by inhibiting the expression of GSDMD. MiR-100-5p in exosomes derived from human umbilical cord mesenchymal stem cells (hUCMSC-Exos) inhibit pyroptosis by inhibiting the expression of FOXO3. HUCMSC-Exos reduce ischemic hypoxia-induced iron death in cardiomyocytes by targeting DMT1. LncRNA-UCA1 in human MSC-Exos and Lnc A2M-AS1 in human BMMSC-Exos inhibit myocardial cell apoptosis by adjusting the miR-873-5p/XIAP axis and miR-556-5p/XIAP axis, respectively.

PCD has a variety of forms, such as apoptosis, autophagy, pyroptosis, and ferroptosis (Figure 2). MSC-Exos can protect the myocardium from ischemic and hypoxic injury by inhibiting apoptosis, autophagy, pyroptosis, and ferroptosis. Exosomes from human umbilical cord-derived MSCs (hUCMSCs) protect cardiomyocytes from acute MI damage and reduce apoptosis by transferring miR-19a to target SOX6, which activates AKT and inhibits JNK3/caspase-3 activation [63]. The PI3K/AKT pathway is an important signaling pathway that regulates apoptosis and survival. Both miR-144 and miR-486-5p in BMMSC-Exos significantly inhibit apoptosis in hypoxic cardiomyocytes by targeting the PTEN/PI3K/AKT signaling pathway [64, 65]. miR-21 in endometrial MSC-Exos can also exert significant antiapoptotic effects by interacting with the PTEN/PI3K/AKT signaling pathway [66]. Similarly, miR-21a-5p and miR-25-3p in BMMSC-Exos can reduce apoptosis in hypoxic cardiomyocytes by downregulating the expression of proapoptotic genes, including FasL, PDPD4, Peli1, and PTEN [67, 68]. In addition, miR-129-5p and miR-338 are enriched in BMMSC-Exos and inhibit myocardial apoptosis and improve cardiac function in MI rats by regulating the TRAF3/NF-*κ*B and MAP3K2/JNK signaling pathways, respectively [69, 70]. Furthermore, Lee et al. [71] found that MSC-derived EVs upregulate the expression of survivin by the miR-199a-3p-AKT-Sp1/p53 signaling pathway, which protects cardiomyocytes from injury in doxorubicin-induced cardiomyopathy.

Autophagy is the process by which lysosomes engulf organelles and other factors to remove unnecessary or dysfunctional cellular components [72]. Recent research has shown that a certain level of autophagy plays a positive role in maintaining the structure and function of cardiomyocytes and that increased autophagy helps protect the heart during myocardial ischemia and hypoxia [73]. Zou et al. [74] found that BMMSC-Exos regulate the postinfarction cardiac microenvironment by promoting autophagy in infarcted hearts. The mechanism of action may be related to increases in the expression of autophagy-related protein 13 in H9c2 cells after MI modeling. It has been found that hUCMSC-Exos attenuate coxsackievirus B3-induced myocarditis by activating the AMPK/mTOR-mediated autophagic flux pathway, thereby attenuating cardiomyocyte apoptosis [75]. Liu et al. [76] found that BMMSC-Exos reduce apoptosis by activating the AMPK/mTOR and AKT/mTOR signaling pathways to induce autophagy in cardiomyocytes. Basal levels of autophagy are critical for cardiac protection, while excessive autophagy promotes cell death and ventricular remodeling [51]. miR-29c and miR-125b in BMMSC-Exos inhibit excessive autophagy by regulating the PTEN/AKT/mTOR and p53/Bnip3 signaling pathways, respectively, thereby reducing the inflammatory response after myocardial I/R injury [77, 78].

The nod-like receptor protein 3 (NLRP3) inflammasome is a multiprotein signaling complex that mediates the maturation of proinflammatory cytokines such as IL-1*β* and IL-18 through interactions with caspase 1 [79]. Pyroptosis is a type of PCD characterized by inflammatory cytokine (caspase-1, NLRP3) release [80, 81]. Tang et al. [81] found that hBMMSC-Exos exhibit significantly reduced expression of the pyroptosis-related proteins caspase-1 and NLRP3, thereby protecting the myocardium from ischemia/reperfusion (I/R) damage. Liang et al. [82] further found that miR-100-5p is enriched in hUCMSC-Exos and inhibits activation of the NLRP3 inflammasome by inhibiting the expression of FOXO3, thus preventing the release of cytokines and blocking pyroptosis. Furthermore, miR-182-5p in BMMSC-Exos can attenuate ischemic myocardial injury and pyroptosis by targeting GSDMD and reducing its expression [83]. DMT1 overexpression promotes ferroptosis induced by hypoxia/reoxygenation (H/R). Song et al. [84] found that hUCMSC-Exos decrease H/R-induced ROS levels, iron deposition, and Fe^2+^ and MDA levels in cardiomyocytes by targeting DMT1. In addition to the above-mentioned models of cell death, cuproptosis is a novel cell death mechanism that is closely related to mitochondrial respiration [85]. Whether the mechanism by which exosomes inhibit cell death is related to cuproptosis has not been confirmed, which may be a new idea for future exosome research.

It has also been found that some lncRNAs are involved in the cardioprotective effects of exosomes. The lncRNA KLF3-AS1 in hMSC-Exos can inhibit the viability, the inflammatory response, and pyroptosis in cardiomyocytes by regulating the miR-138-5p/SIRT1 axis, thus inhibiting the progression of MI [86]. LncRNA-UCA1 in hypoxia-pretreated hMSC-Exos plays a protective role in cardiac injury repair by targeting the miR-873-5p/XIAP axis and increasing the antiapoptotic protein Bcl2 levels [87]. In addition, human BMMSC-Exo-mediated Lnc A2M-AS1 inhibits H/R-induced myocardial injury by the miR-556-5p/XIAP axis [88]. As mentioned above, the cardioprotective effects of stem cell-derived exosomal miRNAs and lncRNAs have been confirmed by in vivo studies in animal models. New therapeutic approaches based on exosomal miRNAs will lay the foundation for the clinical treatment of patients with CVD.

### 4.2. Inhibiting Myocardial Fibrosis

The cardiac repair response after MI can be divided into three stages: inflammation, proliferation, and maturation [89]. Fibroblasts are involved in different stages of heart repair and play different roles in these stages. Fibroblasts exhibit a proinflammatory phenotype during the inflammatory phase and degrade extracellular matrix (ECM) components [90]. At this point, the differentiation of cardiac fibroblasts (CFs) into myofibroblasts is inhibited. When the dead cells are removed, cardiac repair enters the proliferative stage. During this stage, the inflammatory response is suppressed, and most fibroblasts differentiate into myofibroblasts, exhibiting an anti-inflammatory phenotype and producing an ECM that enhances the contractile capacity of the myocardium to maintain the structural and functional integrity of the heart [91]. Therefore, it may be beneficial for the increase of myocardial fibroblasts during the inflammatory period after MI to cardiac repair after MI [89]. Shi et al. [92] found that exosomes derived from hUCMSCs in the inflammatory phase after MI promote the differentiation of fibroblasts into myofibroblasts by increasing the density of myofibroblasts in the infarcted area, thus reducing the inflammatory response of cardiomyocytes. Activation of the Wnt/*β*-catenin signaling pathway is associated with fibrosis in various tissues and organs [93]. It has been found that activation of the Wnt/*β*-catenin signaling pathway can affect myofibroblast generation and promote myocardial repair [94]. Moreover, galectin-3 is a key protein associated with modulation of the Wnt/*β*-catenin signaling pathway. Guo et al. [91] observed in a rat MI model that galectin-3 in hUCMSC-Exos promoted the differentiation of CFs into myofibroblasts in an inflammatory environment by upregulating *β*-catenin levels in fibroblasts. Notably, an appropriate increase in the Wnt signaling pathway can promote the repair of necrotic myocardial tissue after MI, reduce the size of MI, and improve ventricular function [95, 96]. However, persistent overactivation of the Wnt signaling pathway can lead to severe myocardial fibrosis and impair myocardial function [95].

**Figure 3:**
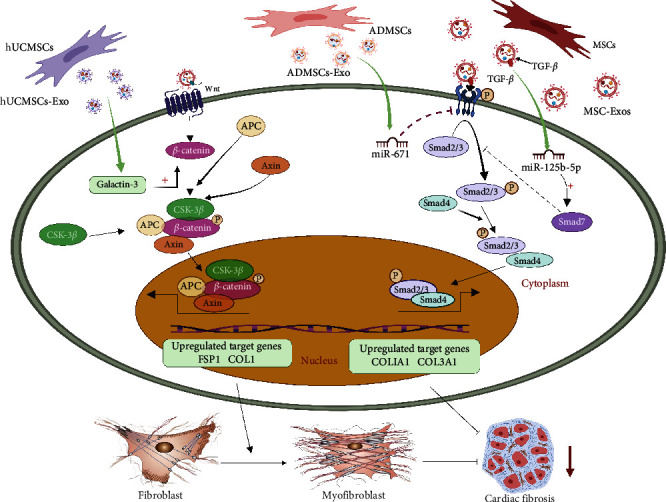
Anti-myocardial fibrosis effect of MSC-Exos. Galactin-3 in exosomes derived from mesenchymal stem cells (MSC-Exos) promotes the differentiation of cardiac fibroblasts into myoblasts in an inflammatory environment by upregulating *β*-catenin levels in fibroblasts. MiR-212-5p and miR-671 in MSC-Exos reduce myocardial infarct-induced fibrosis by regulating the NLRC5/VEGF/TGF-*β*1/Smad axis and TGFBR2/Smad2 axis, respectively. MSC-Exos promote Smad7 expression by inhibiting miR-125b-5p, inhibiting Smad2/3 phosphorylation, and further inhibiting myocardial fibrosis.

Transforming growth factor *β* (TGF-*β*) molecules are multipotent cytokines that cause tissue fibrosis [97]. TGF-*β*1 is essential in most stages of wound healing and scar formation [98]. Smad2 and Smad3 are generally considered the key downstream mediators of TGF-*β*1, and they play a very important role in the expression of matrix collagen and tissue fibrosis [99, 100]. Wu et al. [101] found that BMMSC-Exo-derived miR-212-5p alleviates MI-induced fibrosis by modulating the NLRC5/VEGF/TGF-*β*1/Smad axis. The reduction in TGF-*β*1 directly alleviates ECM deposition and fibrosis. In addition, miR-671 in exosomes derived from adipose-derived mesenchymal stem cell (ADMSC-Exos) can directly target the TGFBR2/Smad2 axis to attenuate myocardial tissue fibrosis in mice with MI, thus alleviating ischemic myocardial injury [102]. Smad7 is an important negative modulator of TGF-*β*/Smad signaling and protects against myocardial damage. It inhibits downstream gene transcription by inhibiting the phosphorylation of Smad2/3 by TGF-*β*1 and interfering with the interactions between receptors and other Smad proteins [103]. Studies have shown that exosomes derived from hUCMSCs can enhance myocardial repair by promoting the expression of Smad7 through inhibition of miR-125b-5p [104] (Figure 3).

Furthermore, Kore et al. [105] found that BMMSC-Exos treatment of MI significantly reduced interstitial and perivascular fibrosis in the ischemic heart and the expression of fibronectin in the infarct and peri-infarct regions; the underlying mechanisms involved suppression of the activation of p-38MAPK and NF-*κ*B, which inhibited fibronectin, collagen 1, and collagen 3 expression. Wang et al. [106] found in a rat myocardial I/R model and an in vitro myocardial microvascular endothelial cell H/R model that BMMSC-Exos promoted microvascular regeneration under stress conditions by regulating platelet-derived growth factor receptor *β* (PDGFR-*β*), thereby alleviating myocardial fibrosis after I/R and ultimately improving cardiac function. They also found that early activation of PDGF-BB/PDGFR-*β* promoted the renewal of functional tissues. By contrast, excessive activation of PDGF-BB/PDGFR-*β* led to fibrosis of functional tissues.

### 4.3. Proangiogenic Effects

Angiogenesis is the physiological process by which new blood vessels form and develop from the existing vascular system. Myocardial angiogenesis after MI is limited. Severe angiogenic dysfunction may lead to systolic dysfunction after heart failure [107]. In a rat model of acute MI, we found that, compared to PBS injection, intramyocardial injection of MSC-EVs significantly enhanced blood flow restoration and reduced infarct size without altering cardiac systolic and diastolic function [61]. In a Sprague‒Dawley rat-induced AMI model, Teng et al. [108] found that compared to PBS, BMMSC-Exos significantly enhanced new functional capillary density and inhibited cell proliferation to impair T-cell function and reduce apoptosis, thus promoting blood flow recovery. Likewise, Xu et al. [17] found that exosomes derived from UCMSCs, ADMSCs, and BMMSCs reduced apoptosis in cardiomyocytes and promoted angiogenesis by increasing the levels of hepatocyte growth factor, angiogenic fibroblast growth factor-*β*, and VEGF in a rat model of MI. The study also showed that the proangiogenic genes Ang1 and Flk1 expression were upregulated in ADMSC-Exo-treated HUVECs, while the antiangiogenic genes Vash1 and TSP1 expression were downregulated [109]. Furthermore, Hu et al. [110] found that human amniotic fluid MSC-Exos promoted angiogenesis by increasing hypoxia-inducible factor 1-*α* (HIF-1*α*) and VEGF expression in rats with isoproterenol-induced cardiac fibrosis. One study in a swine model of MI showed that intramyocardial injection of BMMSC-Exos increased capillary density and blood flow to ischemic myocardial tissue by upregulating the MAPK and AKT/eNOS pathways, resulting in increased cardiac output [111]. In addition, a study on a Yorkshire pig model of metabolic syndrome and chronic myocardial ischemia showed that intramyocardial administration of human BMMSC-Exos increased vascular density and blood flow and improved cardiac function in ischemic myocardial tissue [112]. In a porcine model of MI, we found that placement of decellularized pericardial scaffolds filled with peptide hydrogels and cardiac adipose tissue MSC-EVs on the myocardium resulted in an improvement in cardiac function, a significant increase in right ventricular ejection fraction, marked reductions in myocardial fibrosis and scar tissue, a twofold increase in vascular density, and a reduction in macrophage infiltration observed after 30 days, whereas the expression of anti-inflammatory CD73 was increased by 5.8-fold [113].

**Figure 4:**
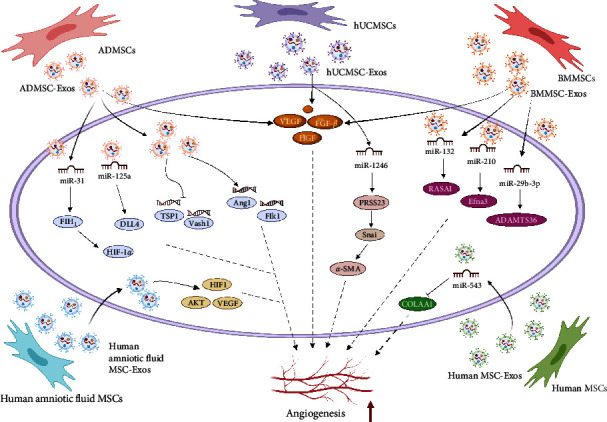
Angiogenic effect of exosomes released by different MSCs after MI. Exosomes derived from umbilical cord mesenchymal stem cells (UCMSCs), adipose-derived mesenchymal stem cells (ADMSCs), and bone marrow mesenchymal stem cells (BMMSCs) promote angiogenesis by increasing levels of hepatocyte growth factor (HGF), angiogenic fibroblast growth factor-*β* (FGF-*β*), and vascular endothelial growth factor (VEGF). Exosomes derived from adipose-derived mesenchymal stem cells (ADMSC-Exos) can upregulate the expression of the angiogenic genes Ang1 (also known as ANGPT1) and Flk1 (also known as KDR) and downregulate the expression of the antiangiogenic genes Vash1 and TSP1 (also known as THBS1). Human amniotic fluid exosomes derived from mesenchymal stem cells (MSC-Exos) promote angiogenesis by increasing the expression of HIF-1*α* and VEGF in fibrotic hearts. Exosomes derived from bone marrow mesenchymal stem cells (BMMSC-Exos) increase capillary density and blood flow in ischemic myocardial tissue by upregulating the MAPK and AKT/eNOS pathways. Extracellular vesicles (EVs) secreted by BMMSCs can promote the angiogenesis of infarcted hearts by regulating the miR-210-Efna3 pathway. MiR-132 mimics and miR-29b-3p loaded in BMMSC-Exos significantly promote neovasculature around the infarcted heart by regulating RASAI gene expression and A Disintegrin and Metalloproteinase with Thrombospondin Motifs 16 (ADAMTS16) expression, respectively. MiR-125a and miR-31 in ADMSC-Exos promote endothelial cell angiogenesis by inhibiting the expression of the angiogenic inhibitor delta-like 4 (DLL4) and activating the FIH1/HIF-1*α* pathways, respectively. MiR-543 in human MSC-Exos promotes the formation of cardiac microvascular endothelial cells by downregulating the expression of COL4A1. miR-1246 in exosomes derived from human umbilical cord mesenchymal stem cells (hUMSC-Exos) promotes angiogenesis by targeting the PRSS23/Snail/*α*-SMA axis.

Wang et al. [114] found that a single intravenous injection of EVs secreted by BMMSCs in mice could promote angiogenesis and improve cardiac function in infarcted hearts. The mechanism by which angiogenesis was promoted may have been related to the miR-210-Efna3 pathway. Transplanting BMMSC-Exos loaded with miR-132 mimics into the ischemic hearts of mice significantly promotes neovascularization around the infarct area by regulating RASAI gene expression [115]. miR-125a and miR-31 in ADMSC-Exos promote endothelial cell angiogenesis by inhibiting the expression of the angiogenic inhibitor delta-like 4 and activating the FIH1/HIF-1*α* pathway, respectively [109, 116]. An increase in miR-29b-3p secreted by BMMSC-Exos can promote angiogenesis and ventricular remodeling in MI rats by targeting A Disintegrin and Metalloproteinase with Thrombospondin Motifs 16 (ADAMTS16) expression [117]. miR-543 in human MSC-Exos promotes proliferation, migration, and angiogenesis in cardiac microvascular endothelial cells through the downregulation of COL4A1 expression [118]. miR-1246 in hUCMSC-Exos promotes HUVEC angiogenesis by targeting the PRSS23/Snail/*α*-SMA axis [119]. These results demonstrate the therapeutic potential of MSC-Exos in ischemic heart disease (Figure 4).

Notably, some studies have shown that although intravenous injection of BMMSC-Exos upregulates some proangiogenic signaling pathway factors, it does not increase the vascular density in the ischemic myocardium, possibly because the effect of intravenous injection on angiogenic signaling is different from that of direct myocardial injection [120]. In addition, the dose of exosomes and the timing of exosome transplantation after MI can affect the outcomes. In conclusion, MSC-Exos undoubtedly exhibit strong potential to improve cardiac function and promote angiogenesis.

### 4.4. Regulating the Microenvironment after MI

**Figure 5:**
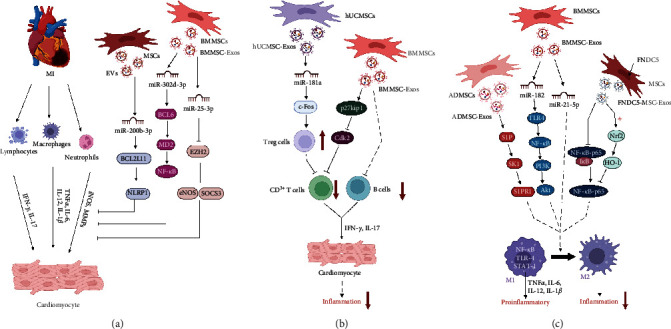
Exosomes secreted by different sources of MSCs regulate the inflammatory microenvironment after myocardial infarction. (a) MiR-200b-3p in extracellular vesicles (EVs) secreted by mesenchymal stem cells (MSCs) inhibits the activation of NLRP1 by inhibiting the expression of Bcl-2-like protein 11 (Bcl2L11), which effectively inhibits the inflammatory response after myocardial infarction (MI). MiR-302d-3p in EVs derived from bone marrow mesenchymal stem cells (BMMSCs) regulates the post-MI inflammatory microenvironment by mediating the BCL6/MD2/NF-*κ*B axis. MiR-25-3p in exosomes derived from bone marrow mesenchymal stem cells (BMMSC-Exos) inhibits the inflammatory response of ischemic myocardial injury by downregulating enhancer of zest homolog 2 (EZH2) and inhibiting suppressor of cytokine signaling 3 (SOCS3) expression. (b) BMMSC-Exos regulate the postinfarction cardiac microenvironment by upregulating p27kip1 and downregulating CDK2 to inhibit the proliferation of CD^3+^ T cells after MI and inhibit the proliferation and differentiation of B cells. MiR-181a in exosomes derived from human umbilical cord mesenchymal stem cells (hUCMSC-Exos) can significantly inhibit the inflammatory response and increase the proportion of Treg cells by targeting the inflammatory transcription factor c-fos, thereby inhibiting T-cell proliferation. (c) MiR-182 in BMMSC-Exos targets the TLR4/NF-*κ*B/PI3K/AKT signaling cascade to promote M1-to-M2 polarization of macrophages. Meanwhile, BMMSC-Exos can promote macrophage differentiation to the M2 phenotype through miR-21-5p, thereby reducing inflammation. Exosomes derived from adipose-derived mesenchymal stem cells (ADMSC-Exos) promote macrophage M2 polarization and cardiac repair by activating S1P/SK1/S1PR1 signal transduction. BMMSC-Exos overexpressing fibronectin type III domain-containing protein 5 (FNDC5) play an anti-inflammatory role by inhibiting the NF-*κ*B signaling pathway and promoting the polarization of M2 macrophages.

Chronic and excessive proinflammatory responses after MI produce adverse left ventricular remodeling [121], which is strongly associated with worsening clinical outcomes after MI; therefore, the management of inflammation after MI is critical for limiting infarct size. Although the entire mechanism of action is not fully understood, it is known that MSC-Exos exert potent immunosuppressive and anti-inflammatory effects [5, 56]. After MI, ATP and NADH depletion is increased, while oxidative stress and cell death are increased [122]. HMSC-Exos can promote cardiac function after I/R injury and reduce myocardial apoptosis by increasing NADH and ATP levels in the I/R heart, reducing oxidative stress, increasing the phosphorylation of GSK-3*β* and AKT, and decreasing the phosphorylation of c-JNK [9]. miR-200b-3p in EVs secreted by MSCs inhibits the activation of NLRP1 by inhibiting the expression of Bcl-2-like protein 11, effectively inhibiting the inflammatory response and oxidative stress in MI mice and improving cardiac function [123]. In addition, Liu et al. [124] found that miR-302d-3p in BMMSC-derived EVs regulates the inflammatory microenvironment by mediating the BCL6/MD2/NF-*κ*B axis to alleviate ventricular remodeling after AMI. In addition, miR-25-3p in BMMSC-Exos inhibits SOCS3 expression by downregulating EZH2 and further inhibits the inflammatory response of the ischemic myocardium [68] (Figure 5(a)).

Studies have shown that MSC-Exos inhibit the invasion and proliferation of immune cells in MI and reduce inflammatory infiltration of myocardial tissue [108]. In addition, the proliferation of CD^3+^ T cells is significantly inhibited following treatment with BMMSC-Exos. The mechanism may be associated with upregulation of p27kip1 and downregulation of CDK2 to induce cell cycle arrest in T cells [125]. Similar results have been reported for previous in vitro research on the interaction of BMMSC-Exos with peripheral blood mononuclear cells. In that study, the researchers found that MSCs induced apoptosis in CD^3+^ T cells and inhibited CpG-stimulated B-cell proliferation and differentiation and the production of IgG, IgA, and IgM [126]. Some studies have shown that BMMSC-Exos treatment significantly reduces the levels of the inflammatory regulators IL-1b, phospho-p38-MAPK, NF-*κ*B, and the NLRP3 inflammasome [105]. Wei et al. [127] found in a mouse myocardial I/R injury model that hUCMSC-Exos overexpressing miR-181a could significantly inhibit the inflammatory response and increase the proportion of Treg cells by targeting the inflammatory transcription factor c-Fos. BMMSC-Exos, which carry miR-125b, restore cardiac function in I/R rats by inhibiting inflammation and apoptosis in I/R cardiomyocytes by targeting SIRT7 [128] (Figure 5(b)).

Macrophages are central mediators of inflammation in the heart and are involved in its development and regression. M1 macrophages show proinflammatory effects and generate inflammatory factors such as IL-6, IL-1*β*, and TNF-*α*, whereas M2 macrophages mediate repair, which is conducive to the activation of CFs, reconstitution of the ECM, and angiogenesis [51, 129]. Zhao et al. [130] demonstrated that miR-182 in BMMSC-Exos targets the TLR4/NF-*κ*B/PI3K/AKT signaling cascade and reduces myocardial I/R damage by polarizing inflammatory macrophages into anti-inflammatory macrophages in the heart. Similarly, Deng et al. [131] found that ADMSC-Exos ameliorate cardiac injury after MI by activating the signaling passway of S1P/SK1/S1PR1 and promoting M2 polarization of macrophages. Another study has shown that intramyocardial injection of BMMSC-Exos in mice with myocardial ischemia can reduce inflammation by promoting the differentiation of macrophages into the M2 phenotype through miR-21-5p, thereby promoting cardiac repair [132]. Correspondingly, BMMSC-Exos overexpressing fibronectin type III domain-containing protein 5 exert anti-inflammatory effects by inhibiting the NF-*κ*B signaling pathway and upregulating the Nrf2/HO-1 axis to promote M2 macrophage polarization [133] (Figure 5(c)). In a mouse dilated cardiomyopathy model, Sun et al. [134] found that MSC-Exos mediated macrophage polarization by modulating the JAK2-STAT6 signaling pathway, thereby improving the inflammatory microenvironment in and reducing inflammatory cell infiltration. Thus, MSC-Exos have a modulatory effect on the microenvironment after MI, which lays an essential foundation for subsequent modification and optimization of MSCs for improved cardioprotective outcomes.

## 5. Pretreatment and Engineering Strategies to Improve the Efficacy of MSC-Derived Exosomes in Cell-Free Therapy

**Figure 6:**
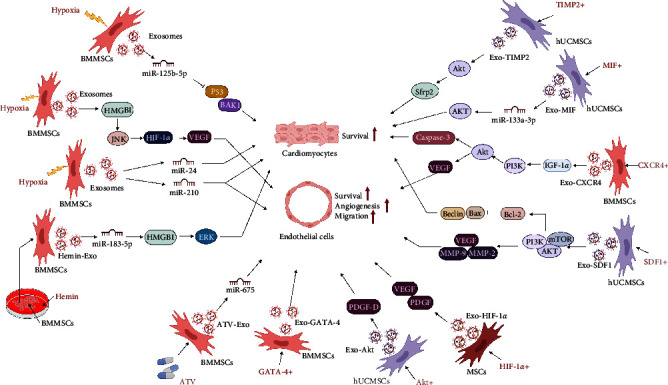
Repair effect of engineered MSC-Exos on ischemic heart. Exosomes secreted by bone marrow mesenchymal stem cells (BMMSCs) preconditioned with moderate hypoxia promote angiogenesis or anti-myocardial apoptosis through upregulation of miR-24, miR-125b-5p, miR-210, HMGB1, etc. HIF-1*α*-overexpressing mesenchymal stem cells (MSCs) secreted exosomes (Exo-HIF-1*α*), SDF1-overexpressing human umbilical cord mesenchymal stem cells (hUCMSCs) secreted exosomes (Exo-SDF1), CXCR4-overexpressing bone marrow mesenchymal stem cells (BMMSCs) secreted exosomes (Exo-CXCR4), TIMP2-modified hUCMSCs secreted exosomes (Exo-TIMP2), and MIF-overexpressing hUCMSCs secreted exosomes (Exo-MIF), AKT-modified hUCMSCs secreted exosomes (Exo-AKT), and GATA-4 overexpressing BMMSCs secreted exosomes (Exo-GATA-4), atorvastatin pretreated BMMSCs secreted exosomes (ATV-Exo), hemin pretreated BMMSCs secreted exosomes (Hemin-Exo) played a better role in promoting angiogenesis or antimyocardial apoptosis in infarcted hearts. HMGB1, high mobilitygroup box 1 protein; SDF1, stromal-derived factor 1; Bax, Bcl-2-associated X protein; Bcl-2, B-Cell CLL/Lymphoma 2; MMP-2, matrix metalloprotein-2; MMP-9, matrix metalloprotein-9; VEGF, vascular endothelial growth factor; AKT, protein kinase B; PDGF-D, platelet-derived growth factor D; HIF-1*α*, hypoxia-inducible factor 1*α*; IGF-1*α*, insulin-like growth factor-1*α*; MIF, macrophage migration inhibitory factor; Sfrp2, secreted frizzled-related protein 2; TIMP2, recombinant tissue inhibitors of metalloproteinase 2; GATA-4, GATA binding protein 4; ATV, atorvastatin.

Exosomes have low immunogenicity, low toxicity, and high engineering potential and are expected to become cell-free therapeutics for a variety of diseases [48]. The biomolecules encapsulated in exosomes fluctuate according to the surrounding microenvironment and the state of MSCs. Although the transplantation of MSC-Exos has shown significant advantages in restoring cardiac function after MI, how to collect more exosomes with strong reparative effects to target the ischemic myocardium effectively still needs to be further explored [135–137]. Many attempts have been made to address this issue. Here, we summarize two possible strategies for improving the therapeutic activity of MSC-Exos: pretreatment and the use of engineered exosomes (Figure 6).

### 5.1. Pretreatment of Exosomes

#### 5.1.1. Hypoxia-Pretreated Exosomes

Hypoxic preconditioning is a common method used in vitro. In general, oxygen tension is considered an essential factor that affects the biological behaviors of stem cell cultures [138, 139]. Several studies have demonstrated that hypoxic preconditioning promotes the survival, proliferation, and migration of MSCs in the context of MI, which enhances the efficacy of transplanted MSCs after MI [140, 141]. The beneficial effect of low oxygen tension on MSCs and the secretion of exosomes from MSCs cultured in a hypoxic environment has gradually attracted extensive attention [142]. Hypoxia preconditioning (0.5% O2 for 24 hr) promotes paracrine proangiogenic effects of BMMSCs, increases vascular density and decreases endogenous cell apoptosis [142]. Zhang et al. [143] found that BMMSC-Exos that were pretreated with hypoxia inhibited apoptosis in cardiomyocytes in AMI rats by upregulating microRNA-24. Similarly, Zhu et al. [144] found that hypoxia-pretreated BMMSC-Exos (1% O2 for 72 hr) inhibited myocardial apoptosis and promoted ischemic heart repair via miR-125b-5p. The mechanism may have involved inhibiting the proapoptotic genes p53 and BAK1 expression. Some studies have shown that hypoxia-pretreated BMMSC-Exos (0.5% O2 for 24 hr) promote miR-210 production and NSMase2 activation via the action of HIF-1*α*, significantly improving the biological characteristics and therapeutic effects of exosomes by increasing vascular density, decreasing cardiomyocyte apoptosis, and reducing fibrosis in the infarcted heart [145]. Gao et al. [146] further confirmed that treatment with hypoxia (5% O2 for 6 days) increased HMGB1 expression in BMMSC-Exos, which activated the JNK/HIF-1*α*/VEGF pathway, thereby promoting angiogenesis in HUVECs. Another study showed that hypoxia-BMMSC-Exos (1% O2 for 48 hr) were more readily absorbed by cells than normal MSC-Exos [147], suggesting that hypoxia treatment can indirectly improve exosome utilization. Therefore, moderate hypoxic pretreatment is a safe, natural, and effective method to optimize the therapeutic effects of MSC-Exos.

#### 5.1.2. Genetically Modified Exosomes

HIF-1*α* is a key transcriptional regulator of the hypoxia response that regulates the expression of many genes, including those encoding angiogenic cytokines [148]. Sun et al. [149] observed that HIF-1*α*-overexpressing exosomes (Exos-HIF-1*α*) exert proangiogenic and cardioprotective effects on the ischemic heart via VEGF- and PDGF-mediated phenotypes and that Exos-HIF-1*α* can rescue angiogenesis, proliferation, and migration in hypoxia-injured HUVECs. Studies have shown that overexpression of SDF1 in hUCMSCs increases the levels of SDF1 in hUCMSC-Exos and can inhibit cardiomyocyte autophagy, promoting endothelial microangiogenesis by activating the PI3K pathway [150]. Exosomes derived from BMMSCs overexpressing the chemokine receptor CXCR4, another major regulator of stem/progenitor cell activities, initiate the signaling pathway of IGF-1/PI3K/AKT in cardiomyocytes, thereby decreasing myocardial apoptosis, promoting angiogenesis, and preventing ventricular remodeling post-MI [151]. Ni et al. [152] found that exosomes derived from TIMP2-modified hUCMSCs can repair the ischemic myocardium by activating the prosurvival AKT/Sfrp2 pathway to inhibit cardiomyocyte apoptosis, remodel the ECM, and promote angiogenesis. Exosomes released by UCMSCs infected with lentiviruses containing macrophage migration inhibitory factor (MIF), a proinflammatory cytokine that is widely expressed in MSCs, show improved cardioprotective effects in promoting angiogenesis and cardiac function and inhibiting apoptosis and fibrosis. The mechanism of MIF-Exos involves miR-133a-3p and the downstream AKT signaling pathway [153]. Liu et al. [154] also confirmed that MIF-overexpressing BMMSC-Exos inhibit mitochondrial fission induced by hypoxia and serum deprivation by activating the AMPK signaling pathway, which can reduce apoptosis and mitochondrial fragmentation of cardiomyocytes and promote cardiac function. Ma et al. [155] found that exosomes derived from AKT-modified hUCMSCs secreted more platelet-derived growth factor D to promote angiogenesis and improve cardiac function in mice than hUCMSC-Exos. Moreover, He et al. [156] found that exosomes secreted by GATA-4-expressing BMMSCs could increase myocardial vascular density and improve cardiac function in a mouse MI model. These reports suggest that genetic modifications can effectively increase the overall functional performance of MSC-Exos in MI injuries. These preclinical findings thus provide an important foundation for clinical application and transgene-optimized exosome transduction.

#### 5.1.3. Drug-Pretreated Exosomes

MSC-Exos that are pretreated with drugs or cytokines have also been shown to have excellent cardioprotective effects. Huang et al. [157] demonstrated that exosomes derived from BMMSCs that were pretreated with atorvastatin (BMMSCATV-Exos) exhibited an improved ability to enhance angiogenesis and protect cardiomyocytes while improving cardiac function following infarction. Mechanistically, lncRNA H19 in BMMSCATV-Exos activated the expression of miR-675 and promoted angiogenesis. Some studies have shown that hBMMSC-Exos pretreated with hemin are superior to nonpretreated hBMMSC-Exos in improving cardiac function after infarction, and miR-183-5p enriched in hemin-pretreated MSC-Exos inhibits ischemia-induced cardiomyocyte senescence via inhibition of the HMGB1/ERK pathway [158]. In a murine model of MI, Xu et al. [159] observed that pretreatment of BMMSC-Exos with low concentrations of LPS promoted M2 macrophage polarization in vitro and attenuated postinfarct inflammation and cardiomyocyte apoptosis through mediation of macrophage polarization. These drug-pretreated exosomes have been shown to potentially mitigate transplant rejection, providing a strong basis for improved functioning of exosomes in vivo.

In addition, exosomes derived from hUCMSCs encapsulated in functional peptide hydrogels can augment myocardial function by decreasing inflammation, fibrosis, apoptosis, and angiogenesis of the adhesions [160]. Various in vitro exosome pretreatment methods have improved the transplantation rate and survival of exosomes in vivo, providing more possibilities for future MI treatment.

### 5.2. Exosome Targeting

In the previous section, we described the beneficial effects of MSC-Exos on the heart. However, accurate targeting of exosomes to recipient cells is still faced with serious challenges. Homing peptides or ligand fragments discovered by in vivo biopanning methods and phage display with fusion to enriched molecules outside exosomes have been used to improve the ability of exosomes carrying cognate receptors to target specific tissues or organs. Exosomal surface ligands or homing peptides improve drug delivery targeting and efficiency [161]. Wang et al. [162] demonstrated that the ischemic myocardial targeting peptide CSTSMLKAC can directly target the ischemic myocardium, thereby increasing the targeting and utilization of exosomes. In another study, Vandergriff et al. [163] performed targeted injection of exosomes bound to the cardiac homing peptide into infarcted hearts and found that exosome retention increased in the cardiac sections of isolated rat cardiomyocytes and promoted functional recovery in animal models by inducing cardiomyocyte proliferation, reducing fibrosis, and promoting angiogenesis. The cardiac-targeting peptide (CTP)-Lamp2b has been modified to yield exosomes expressing CTP-Lamp2b on the exosome membrane (CTP-Exos). Compared with native exosomes, CTP-Exos deliver exosomes to heart cells and cardiac tissues significantly more effectively [164]. Moreover, peptide libraries and phage displays have identified many peptides located in the cardiovascular system, such as normal cardiomyocytes, myocardial cells injured by I/R, heart failure, atherosclerotic plaques, and vasculature [165]. Targeted peptide-modified MSC-Exos undoubtedly provide new possibilities with which to improve the efficiency of targeted therapy for MI.

## 6. Human Induced Pluripotent Stem Cell-Derived Exosomes in MI

The limited sources of MSCs have greatly hindered clinical research and applications. In recent years, MSCs derived from induced pluripotent stem cells (iMSCs) have attracted widespread attention. Studies have found that induced pluripotent stem cells (iPSCs) show similar capacity and morphology to ESCs for self-renewal and differentiation without ethical concerns [166, 167]. Compared with MSCs, iMSCs have been shown to have greater advantages in immunomodulation, microenvironmental regulation, and secretion of bioactive factors [167]. In addition, iMSCs have better proliferation ability and lower immunogenicity than MSCs [168]. Notably, studies have shown that the donor age of MSCs plays an important role in regenerative capacity, where MSCs from young donors have better regenerative capacity than those from older donors. By contrast, iMSCs can bypass tissue- and age-related heterogeneity problems [169, 170]. The therapeutic potential of iMSC-derived exosomes in ischemic heart disease has been heavily investigated. Gao et al. [136] found in an animal infarction model that exosomes from human iPSC-derived cardiomyocytes were also cardioprotective and that they improved the recovery of the ischemic myocardium without increasing the incidence of arrhythmic complications. In addition, exosomes from iMSCs ameliorate myocardial injury induced by severe acute pancreatitis through activation of the AKT/Nrf2/HO-1 axis [166]. Another study has shown that iMSC-derived exosomes regulate autophagy by regulating the PI3K-AKT-mTOR and MAPK signaling pathways to improve cardiac function after MI [171]. These exosomes also play very significant roles in the regulation of apoptosis, inflammation, fibrosis, and angiogenesis [172–173]. Currently, the study of exosomes in CVD is progressing toward iMSC-derived exosomes and enriched miRNA candidates. It is promising to use iMSC-derived exosomes under certain conditions. However, the limited amount of evidence for clinical applications and the absence of evidence at the clinical trial level means that research on iMSC-derived exosomes is still at a nascent stage. The clinical use of iMSC-derived exosomes in the treatment of CVD still needs to be investigated in further detail.

## 7. Clinical Trial of MSCs in the Treatment of MI

To date, there is abundant preclinical evidence for the efficacy of exosomes in animal models of MI [112, 151]. MSC-Exos have shown great potential in numerous studies [60, 61]. The safety of exosomes has been tentatively demonstrated in certain clinical trials, although most exosome studies have been preclinical [174]. In addition, there are already clinical trials with MSC-Exos for the treatment of MI that are in the recruitment stage (NCT05669144), which means that new data on immunogenicity should be available soon. The safety and efficacy of exosome therapy still need to be confirmed by further clinical studies. Although the results of clinical studies on the use of MSC-Exos for the treatment of MI or other heart-related problems have not yet been published, clinical studies on the use of MSC-Exos in the treatment of other systemic diseases, including cerebrovascular disorders, diabetes mellitus type 1, and Alzheimer's disease, are already underway (see Table 1).

## 8. Conclusion

In this review, we investigated the therapeutic effects of exosomes on MI. As cell-free substitutes for stem cell therapy, exosomes have the potential to improve myocardial fibrosis and apoptosis, regulate the cardiac microenvironment after infarction, promote myocardial regeneration, and increase neovascularization after I/R injury. Although recent studies have demonstrated the outstanding performance of exosomes in attenuating myocardial injury, the specific effects of exosome cargoes on particular signaling pathways need to be further explored. Meanwhile, due to the limitations of exosome extraction technology in the past, most of the early studies on the mechanism of MSC-Exo-mediated myocardium repair were carried out via miRNA-related sequencing analysis or detection; relatively few studies involved the detection of proteins, lipids, and other components. The mechanisms of action of the other components of exosomes in the treatment of infarction need to be further studied. Moreover, the heterogeneity of donor cells, the cell growth environment in vitro, and the low targeting and low retention rates of exosomes in recipient cells may affect the function of exosomes. Engineering technology for exosomes makes them natural nanocarriers for the delivery of molecular drugs, as well as through surface modification enhances the target specificity of exosomes, increasing their value for clinical applications. However, exosomes obtained by pretreating parental cells are usually less efficient in terms of drug loading, and the drug levels may be nonuniform. In contrast, direct loading of drugs into exosomes enables better control of drug loading. The development of engineered exosomes is still in the preliminary stage. Although, as drug carriers, the effectiveness of exosomes has been preliminarily established in preclinical studies, further studies are still needed to verify their long-term safety in the future. In addition, standardized methods of exosome isolation and purification need to be further explored. The cardiac muscle repair ability of exosomes obtained from different MSC sources may vary, but there are few studies that are conclusive as to which type of MSC source is most effective and suitable for clinical use. The nature of MSCs depends largely on their tissue origin and the conditions of cellular culture, which suggests that the most effective types of transplanted cells should be selected according to disease characteristics and the characteristics of the different MSCs in future clinical use. Thus, in future clinical applications, the most effective types of transplanted cells should be chosen according to the characteristics of the disease and the different MSCs. We believe that addressing these challenges will lead to the widespread application of exosomes in clinical settings, thereby revealing new clinical strategies for exosomes and enabling the exploration of new clinical therapeutic approaches to benefit patients with MI.

## Figures and Tables

**Table 1:** Mesenchymal stem cell-derived exosomes in clinical trials.

NCT number	Condition or disease	Status	Phase	Brief summary	Sponsor
NCT03384433	Cerebrovascular disorders	Unknown status	Phase 1, Phase 2	Allogenic mesenchymal stem cell-derived exosome in patients with acute ischemic stroke	Isfahan University of Medical Sciences

NCT05813379	Antiaging	Recruiting	Phase 1, Phase 2	Mesenchymal stem cells derived exosomes in skin rejuvenation	Isfahan University of Medical Sciences

NCT04544215	Drug-resistant	Unknown status	Phase 1, Phase 2	A clinical study of mesenchymal progenitor cell exosomes nebulizer for the treatment of pulmonary infection	Ruijin Hospital

NCT05871463	Decompensated liver cirrhosis	Recruiting	Phase 2	Effect of mesenchymal stem cells-derived exosomes in decompensated liver cirrhosis	Research Institute for Gastroenterology and Liver Diseases (RIGLD)

NCT05523011	Psoriasis	Completed	Phase 1	Safety and tolerability study of MSC exosome ointment	Paracrine Therapeutics Dermatology Pte. Ltd

NCT04356300	Multiple organ failure	Not yet recruiting	Not applicable	Exosome of mesenchymal stem cells for multiple organ dysfunction syndrome after surgical repair of acute type A aortic dissection	Fujian Medical University

NCT05261360	Knee; injury, meniscus (lateral) (medial)/meniscus tear/meniscus lesion/6 more	Recruiting	Phase 2	Clinical efficacy of exosome in degenerative meniscal injury	Eskisehir Osmangazi University

NCT05499156	Perianal fistula in patients with Crohn's disease	Unknown status	Phase 1, Phase 2	Safety of injection of placental mesenchymal stem cell-derived exosomes for treatment of resistant perianal fistula in Crohn's patients	Tehran University of Medical Sciences

NCT04276987	Coronavirus	Completed	Phase 1	A pilot clinical study on inhalation of mesenchymal stem cells exosomes treating severe novel coronavirus pneumonia	Ruijin Hospital

NCT05808400	Long COVID-19 syndrome	Recruiting	Early Phase 1	Safety and efficacy of umbilical cord mesenchymal stem cell exosomes in treating chronic cough after COVID-19	Huazhong University of Science and Technology

NCT05402748	Fistula perianal	Recruiting	Phase 1, Phase 2	Safety and efficacy of injection of human placenta mesenchymal stem cells derived exosomes for treatment of complex anal fistula	Tehran University of Medical Sciences

NCT04313647	Healthy	Completed	Phase 1	A tolerance clinical study on aerosol inhalation of mesenchymal stem cells exosomes in healthy volunteers	Ruijin Hospital

NCT05413148	Retinitis pigmentosa	Recruiting	Phase 2, Phase 3	The effect of stem cells and stem cell exosomes on visual functions in patients with retinitis pigmentosa	TC Erciyes University

NCT05216562	SARS-CoV2 infection	Recruiting	Phase 2, Phase 3	Efficacy and safety of EXOSOME-MSC therapy to reduce hyperinflammation in moderate COVID-19 patients (EXOMSC-COV19)	Dermama Bioteknologi Laboratorium

NCT03437759	Macular holes	Unknown status	Early Phase 1	MSC-Exos promote healing of MHs (MSCs)	Tianjin Medical University

NCT04602104	Acute respiratory distress syndrome	Unknown status	Phase 1, Phase 2	A clinical study of mesenchymal stem cell exosomes nebulizer for the treatment of ARDS	Ruijin Hospital

NCT04213248	Dry eye	Recruiting	Phase 1, Phase 2	Effect of UMSCs derived exosomes on dry eye in patients With cGVHD	Zhongshan Ophthalmic Center, Sun Yat-sen University

NCT05669144	Myocardial infarction/myocardial ischemia/myocardial stunning	Recruiting	Phase 1, Phase 2	Cotransplantation of mesenchymal stem cell-derived exosomes and autologous mitochondria for patients candidate for CABG surgery	Tehran University of Medical Sciences

NCT05787288	COVID-19 pneumonia	Recruiting	Early Phase 1	A clinical study on the safety and effectiveness of mesenchymal stem cell exosomes for the treatment of COVID-19	First Affiliated Hospital of Wenzhou Medical University

NCT02138331	Diabetes mellitus type 1	Unknown status	Phase 2, Phase 3	Effect of microvesicles and exosomes therapy on *β*-cell mass in type I diabetes mellitus (T1DM)	General Committee of Teaching Hospitals and Institutes, Egypt

NCT04173650	Dystrophic epidermolysis bullosa	Not yet recruiting	Phase 1, Phase 2	MSC EVs in dystrophic epidermolysis bullosa	Aegle Therapeutics

NCT05354141	Acute respiratory distress syndrome/ARDS	Recruiting	Phase 3	Extracellular vesicle treatment for acute respiratory distress syndrome (ARDS) (EXTINGUISH ARDS)	Direct Biologics, LLC

NCT04493242	COVID-19 ARDS	Completed	Phase 2	Extracellular vesicle infusion treatment for COVID-19-associated ARDS	Direct Biologics, LLC

NCT04998058	Bone loss, osteoclastic/bone loss, alveolar/alveolar bone loss/2 more	Not yet recruiting	Phase 1, Phase 2	Autogenous mesenchymal stem cell culture-derived signaling molecules as enhancers of bone formation in bone grafting	Pontificia Universidade Católica do Rio Grande do Sul

NCT05387278	COVID-19 acute respiratory distress syndrome/respiratory distress syndrome	Recruiting	Phase 1	Safety and effectiveness of placental derived exosomes and umbilical cord mesenchymal stem cells in moderate to severe acute respiratory distress syndrome (ARDS) associated with the novel coronavirus infection (COVID-19)	Vitti Labs, LLC

NCT04388982	Alzheimer disease	Unknown status	Phase 1, Phase 2	The safety and the efficacy evaluation of allogenic adipose MSC-Exos in patients with Alzheimer's disease	Ruijin Hospital

*Note*. Searched by ClinicalTrials.gov (https://clinicaltrials.gov/, accessed on 1 October 2023).
